# Mesenchymal stromal cells secretome restores bioenergetic and redox homeostasis in human proximal tubule cells after ischemic injury

**DOI:** 10.1186/s13287-023-03563-6

**Published:** 2023-12-10

**Authors:** João Faria, Sandra Calcat-i-Cervera, Renata Skovronova, Bonnie C. Broeksma, Alinda J. Berends, Esther A. Zaal, Benedetta Bussolati, Timothy O’Brien, Silvia M. Mihăilă, Rosalinde Masereeuw

**Affiliations:** 1 Division of Pharmacology, Department of Pharmaceutical Sciences, Utrecht Institute for Pharmaceutical Sciences, Utrecht University, Universiteitsweg 99, 3584 CG Utrecht, The Netherlands; 2 College of Medicine, Nursing and Health Science, School of Medicine, Regenerative Medicine Institute (REMEDI), University of Galway, Galway, Ireland; 3 Department of Molecular Biotechnology and Health Sciences, University of Turin, Turin, Italy; 4 Danone Nutricia Research, Utrecht, The Netherlands; 5 Division of Cell Biology, Metabolism and Cancer, Department of Biomolecular Health Sciences, Faculty of Veterinary Medicine, Utrecht University, Utrecht, The Netherlands; 6 CÚRAM, SFI Research Centre for Medical Devices, University of Galway, Galway, Ireland

**Keywords:** Ischemia, Proximal tubule, Mesenchymal stromal cell, Extracellular vesicles, Metabolism

## Abstract

**Background:**

Ischemia/reperfusion injury is the leading cause of acute kidney injury (AKI). The current standard of care focuses on supporting kidney function, stating the need for more efficient and targeted therapies to enhance repair. Mesenchymal stromal cells (MSCs) and their secretome, either as conditioned medium (CM) or extracellular vesicles (EVs), have emerged as promising options for regenerative therapy; however, their full potential in treating AKI remains unknown.

**Methods:**

In this study, we employed an in vitro model of chemically induced ischemia using antimycin A combined with 2-deoxy-d-glucose to induce ischemic injury in proximal tubule epithelial cells. Afterwards we evaluated the effects of MSC secretome, CM or EVs obtained from adipose tissue, bone marrow, and umbilical cord, on ameliorating the detrimental effects of ischemia. To assess the damage and treatment outcomes, we analyzed cell morphology, mitochondrial health parameters (mitochondrial activity, ATP production, mass and membrane potential), and overall cell metabolism by metabolomics.

**Results:**

Our findings show that ischemic injury caused cytoskeletal changes confirmed by disruption of the F-actin network, energetic imbalance as revealed by a 50% decrease in the oxygen consumption rate, increased oxidative stress, mitochondrial dysfunction, and reduced cell metabolism. Upon treatment with MSC secretome, the morphological derangements were partly restored and ATP production increased by 40–50%, with umbilical cord-derived EVs being most effective. Furthermore, MSC treatment led to phenotype restoration as indicated by an increase in cell bioenergetics, including increased levels of glycolysis intermediates, as well as an accumulation of antioxidant metabolites.

**Conclusion:**

Our in vitro model effectively replicated the in vivo-like morphological and molecular changes observed during ischemic injury. Additionally, treatment with MSC secretome ameliorated proximal tubule damage, highlighting its potential as a viable therapeutic option for targeting AKI.

**Supplementary Information:**

The online version contains supplementary material available at 10.1186/s13287-023-03563-6.

## Background

Ischemia/reperfusion injury (IRI) is a leading cause of acute kidney injury (AKI) and can even result in kidney failure after transplantation. Current treatments mainly support or replace kidney function, with few effective strategies to prevent or treat ischemic AKI [1, 2]. During ischemia, the temporary restriction in blood flow to the kidney generates a local hypoxic environment, thereby decreasing ATP production and altering mitochondrial metabolism. When blood flow is restored, oxygen levels rapidly increase, mitochondrial oxidative phosphorylation and the production of reactive oxygen species (ROS) increase leading to oxidative stress and inflammation, which exacerbate further tissue damage [3, 4].

Within the kidney, proximal tubule epithelial cells (PTECs) are particularly susceptible to IRI given their high active metabolism, supported by abundant mitochondria present, dependence on aerobic oxidative phosphorylation [5], and limited ability to undergo anaerobic glycolysis [6]. Severe IRI can cause permanent tissue damage and functional impairment; therefore, it is important to understand the underlying mechanisms and develop therapeutic interventions.

In recent years, cell-based regenerative therapies have been explored in a wide range of nephropathies to facilitate tissue repair following kidney injury. In particular, mesenchymal stromal cells (MSCs) have shown promise by promoting angiogenesis, tubular cell turnover, and modulation of immune response and inflammation in preclinical [7–9] and clinical research [10, 11]. Lately, it has been reported that MSCs also exert antioxidant properties that may enhance their cytoprotective effects in IRI, such as damping mitochondrial dysfunction [12] and increasing cell bioenergetics [13], ultimately reducing oxidative stress [12–16]. These such renoprotective mechanisms are suggested to be mediated through paracrine signaling, which involves cytokines, growth factors, and nucleic acids, often harbored in extracellular vesicles (EVs) [14–18]. However, the precise mechanism is still unknown, partially due to the inherent heterogeneity of the MSCs related to the various sources of origin [19–22]. Importantly, a comparison of efficiency between various MSCs to attenuate kidney damage after IRI remains to be explored.

To address this, we established an in vitro model to replicate the effects of ischemia on PTECs by suppressing mitochondrial respiration and glycolysis under hypoxia. We assessed various parameters of mitochondrial function, including metabolic activity, mitochondrial mass, mitochondrial membrane potential, metabolic profile, and cytoarchitecture as well as the cell metabolome. Afterwards cells were treated with MSC secretome isolated from adipose tissue (A), bone marrow (B), and umbilical cord (U) to determine whether mitochondrial function and metabolic activity could be restored during the reperfusion phase.

## Materials and methods

### Cell culture

#### ciPTEC-14.4

The ciPTEC-14.4 cell line (RRID: CVCL_W184) was purchased from Cell4Pharma (Oss, the Netherlands), obtained at passage 38 and cultured as reported previously [23]. Mycoplasma contamination was checked monthly and was found to be negative in all cells used. ciPTECs were used for experiments from passage numbers 42–50, during which robust and reproducible results were obtained in agreement with previous findings [24–26].

## MSCs culture

The MSCs, all human-derived, were obtained within the European Union’s Horizon 2020 research and innovation collaborative network RenalToolBox (Grant Agreement 813839) and well-characterized [27]. A-MSCs from lipoaspirates were processed in Heidelberg (Germany) after obtaining informed consent (Mannheim Ethics Commission; vote number 2006-192NMA). B-MSCs provided by Galway (Ireland) (Galway University Hospital Clinical Research Ethics Committee; approval number 02/08) were purchased from Lonza (Basel, Switzerland), and U-MSCs with informed consent obtained in accordance with the Declaration of Helsinki were sourced from the NHS Blood and Transplant and transferred to the University of Liverpool (United Kingdom) (NHS Blood and Transplant, Cellular and Molecular Therapies; approval number: RTB21112019). MSCs were cultured as reported previously [27]. Briefly, A-MSCs were seeded at a density of 300 cells/cm^2^ and B- and U-MSCs at 3000 cells/cm^2^ at 37 °C with 5% (v/v) CO_2_, and cultured in basic growth medium (MEM-⍺ media, Gibco, ThermoFisher Scientific), supplemented with 10% of fetal bovine serum (FBS, Gibco, ThermoFisher Scientifics, Cat-No. 10,270–106, Lot 42Q7096K) until reaching 80% confluency. A thorough characterization of all three MSC sources, including morphology, growth kinetics, differentiation capabilities, and immunophenotypic profiles, was recently published [27]. In the current study, all MSCs were cultured from passage numbers 3–6.

### CM collection

Upon reaching 80% confluency, MSCs were washed once with HBSS and incubated for 24 h in serum-free MEM-⍺ media. The supernatant was then collected and centrifuged for 5 min at 400*g* to remove cell debris before being transferred to Amicon Ultra-15 centrifugal units (3 kDa molecular cutoff) (Millipore, UFC900324) for concentration at 3000*g* for 90 min at 4 °C. Concentrated CM was then stored at − 80 °C until further use.

### EV isolation

EVs from MSCs were collected as reported previously [28]. Briefly, the supernatant was collected and centrifuged for 10 min at 300*g* to remove cell debris, transferred into new tubes and centrifuged for 20 min at 3000*g* to discard apoptotic cells. The supernatant was then ultracentrifuged for 1 h at 10,000*g* and 4 °C, using the Beckman Coulter Optima L-100K Ultracentrifuge with the rotor type 70 Ti. At this speed, the subpopulation of 10 k EVs (large EVs, LEVs) was collected. The supernatant was further ultracentrifuged for 1 h at 100,000*g* and 4 °C, to obtain the 100 k EV (small EVs, SEVs) subpopulation. The EV pellet was resuspended in PBS supplemented with 0.1% DMSO and stored at − 80 °C until further use.

### ciPTEC ischemic model

To mimic ischemia in vitro, ciPTECs were chemically exposed to 10 nM of antimycin A (AA, Sigma-Aldrich, A8674) and 20 mM of 2-deoxy-D-glucose (2DG, Sigma-Aldrich D6134), as previously described [29–32], for 24 h in serum-free medium (SFM) at 37 °C under normoxia (21% O_2_). Additionally, to enhance the simulation of blood supply disruption which deprives cells of nutrients, we exposed these chemically treated ciPTECs to an hypoxic environment (1% O2). The experimental groups were labeled as: sham, ciPTECs cultured in serum-containing medium; vehicle, ciPTECs cultured in SFM; and ischemia, ciPTECs exposed to AA and 2DG in SFM and under normoxia (21% O_2_) or hypoxia (1% O_2_).

Afterward, ischemic cells were washed once with Hank’s balanced salt solution (HBSS; Gibco, Life Technologies) to remove AA and 2DG before being treated with MSC secretome, either as conditioned medium (CM) or EVs (small, SEV; and large, LEV) [28] from each of the MSCs sources for an additional 24 h at 37 °C. When collecting MSC bioproducts for both the secretome and the EVs, the generating MSCs were harvested and counted to calculate the cell-equivalent concentration of the secretome and EVs. This was used to enable the comparison of the effects exerted by both bioproducts under cell-equivalent doses. Based on previous studies reported in the literature [33, 34], we administered the amount of bioproduct equivalent obtained from 2 MSCs per each ciPTEC in SFM (ratio 2:1). This study incorporated three donors for each MSC type, and the average values from these three donors were employed for subsequent in-depth analysis.

To effectively assess the efficacy of MSC secretome in mitigating ischemic damage, we established a comparative analysis. On one hand, we exposed ischemic ciPTECs to MSC secretome in SFM, to allow us the study of the specific effects of the secretome. On the other hand, we subjected ischemic ciPTECs to serum-containing medium, replicating the conditions in which blood flow is reintroduced post-ischemia (labeled as ‘reperfusion’ group). By comparing the effects of MSC secretome treatment under serum-free conditions with those in a reperfusion-like setting, we aimed to gain insights into the potential benefits of the secretome.

### Injury and treatment assessment

The same readouts performed to assess the ischemic injury were repeated to determine MSC secretome therapeutic effect. All experiments were performed on 96-well plates (Greiner Bio-One, Frickenhausen, Germany), unless stated otherwise.

### Cell metabolic activity

Cell metabolic activity was measured using PrestoBlue® reagent (ThermoFischer Scientific, A13262) [35]. ciPTECs were rinsed once with HBSS and incubated with PrestoBlue® (diluted 1:10 in SFM), in the dark. After 1 h of incubation at 37 °C, the fluorescence was measured using the GloMax® Discover microplate reader (Promega, Wisconsin, United States), at an excitation wavelength of 530 nm and emission wavelength of 590 nm.

### ATP production

ATP production was quantified using the CellTiter-Glo® 2.0 reagent (Promega, G9242) [36]. ciPTECs were incubated with 100 μl/well of reagent, and the solutions were mixed on an orbital shaker for 2 min. The plates were incubated at room temperature for 10 min, and the luminescent signal was read using the GloMax® Discover microplate reader.

### Intracellular ROS production

The generation of intracellular ROS was measured using a cell permeant fluorogenic substrate, CM-H_2_DCFDA (Invitrogen, C6827) [37]. ciPTECs were rinsed once with HBSS before being loaded with CM-H_2_DCFDA (50 μM in SFM medium) and incubated at 37 °C in the dark for 20 min. H_2_O_2_ (500 μM) was used as a positive control. Following the incubation period, cells were washed twice with HBSS and lysed for 10 min in 0.1 M NaOH. Lastly, fluorescence was measured at excitation/emission wavelengths of 492/518 nm, using the GloMax® Discover microplate reader.

### Mitochondrial mass

The mitochondrial mass was quantified using MitoTracker™ Orange CMTMRos (Invitrogen, M7510) [38]. ciPTECs were rinsed once with HBSS, loaded with MitoTracker Orange CMTMRos (200 nM in SFM), and incubated at 37 °C in the dark for 30 min. Additionally, ciPTECs were counterstained with 1 μM Hoechst 33,342 for nuclei detection. Afterward, ciPTECs were washed with HBSS, and fluorescence was measured using the GloMax® Discover microplate reader at excitation/emission wavelengths of 554/576 for MitoTracker™ Orange CMTMRos and 361/497 for Hoechst. Fluorescent values were corrected to account for background signals. Subsequently, the quantification of mitochondrial mass was executed by calculating the ratio of fluorescent values derived from MitoTracker™ Orange CMTMRos to those originating from Hoechst.

### Mitochondrial membrane potential (Δψ)

The integrity of mitochondrial membrane potential (Δψ) was measured using JC-10 (Abcam, ab112134) [39]. ciPTECs were rinsed once with HBSS and incubated with 50 μl of JC-10 loading solution for 30 min at 37 °C in the dark. Subsequently, 50 μl of assay buffer B was added to the plate, and fluorescence was read at excitation/emission wavelengths of 490/525 and 540/590 for ratio analysis, using the GloMax® Discover microplate reader. In the assay, carbonyl cyanide 4-(trifluoromethoxy) phenylhydrazone (FCCP, 10 µM), a mitochondrial uncoupler, was used as a positive control for loss of Δψ.

### Bioenergetic profile

The oxygen consumption rate (OCR) and extracellular acidification rate (ECAR) were monitored in real-time using the Seahorse extracellular flux analyzer XF 96 (Agilent Technologies, USA). ciPTECs were seeded into a XF96 cell culture microplate (Seahorse Bioscience) at a density of 4000 cells/well. Prior to starting the assay, cells were incubated in a non-CO_2_ incubator at 37 °C. Bioenergetic profiles were measured by serial injections of oligomycin (1 µM), FCCP (2 µM) and rotenone/antimycin A (Rot/AA, 0.5 µM, respectively) with the Seahorse XF Cell Mito Stress Test [40]. At the end of the assay, cells were lysed in RIPA buffer (ThermoFisher Scientific, 89,900) and the protein content per well was determined using the Pierce™ BCA Protein Assay Kit (ThermoFisher Scientific, 23227).

The OCR and ECAR values were normalized to the protein values, and subsequently, several mitochondrial-related parameters were extracted. Following the calculations described by Mookerjee et al. [41, 42], OCR and ECAR were converted to the same units (pmol ATP/min/µg protein) and used to calculate net rates of ATP that can originate from between glycolysis (J_ATPglyc_) and oxidative phosphorylation (J_ATPox_). J_ATPox_ can be further represented as ATP production due to the tricarboxylic acid cycle (J_ATPox TCA_) and rate coupled to oxidative phosphorylation (J_ATPox coupled_).

### Immunofluorescence analysis

For immunofluorescence procedures, ciPTECs were cultured in a 96-well black/*clear* bottom plates (ThermoFischer, 165305) [35]. At the end of the experimental protocol, cells were washed with HBSS and fixed in 4% paraformaldehyde (ThermoFisher Scientific) for 10 min at room temperature (RT). After fixation, ciPTECs were permeabilized with 0.3% Triton X-100 in PBS and blocked in blocking buffer (2% FBS, 2% BSA, 0.1% Tween20 in PBS). Primary and secondary antibodies were incubated for 1 h at RT, and samples were washed three times in 0.1% Tween in PBS for 5 min. Nuclei were stained using 4′,6-diamidino-2-phenylindole (DAPI) for 7 min, followed by three washing steps in 0.1% Tween in PBS for 5 min. Immunofluorescence was conducted using confocal microscopy (Leica TCS SP8 X) and the software Leica Application Suite X. The antibodies are listed in Additional file 7: Table S1.

### Metabolomics analysis

LC–MS analysis was performed on a Q-Exactive HF mass spectrometer (Thermo Scientific) coupled to a Vanquish autosampler and pump (Thermo Scientific). Metabolites were separated using a Sequant ZIC-pHILIC column (2.1 × 150 mm, 5 μm, guard column 2.1 × 20 mm, 5 μm; Merck) with acetonitrile and eluent A [20 mM (NH4)2CO3, 0.1% NH4OH in ULC/MS grade water (Biosolve)]. The gradient ran from 20% eluent to 60% eluent in 20 min, followed by a wash step at 80% and equilibration at 20%. The flow rate was set at 100 μl/min. The MS operated in polarity-switching mode with spray voltages of 4.5 kV and − 3.5 kV. Metabolites were identified and quantified on the basis of exact mass within 5 ppm and further validated by concordance with retention times of standards, and peak areas were normalized based on total signal [43]. Analysis was performed using TraceFinder software (Thermo Scientific), R and MetaboAnalyst software.

### Statistical analysis

Quantitative data are reported as mean ± standard deviation (SD). N indicates the number of biological replicates and *n* the number of independent experiments. In fluorescence and luminescence-based assays, data were corrected for background, normalized to untreated cells, and presented as a fold-change to the control group. Statistical analyses were performed using GraphPad Prism version 9.2.0 (GraphPad Software, Inc., San Diego, CA, USA). The statistical test and replicates are indicated in the figure legends. A *p* value < 0.05 was considered statistically significant.

## Results

### Treatment with MSCs secretome restores cell morphology upon ischemia-induced damage

**Fig. 1:**
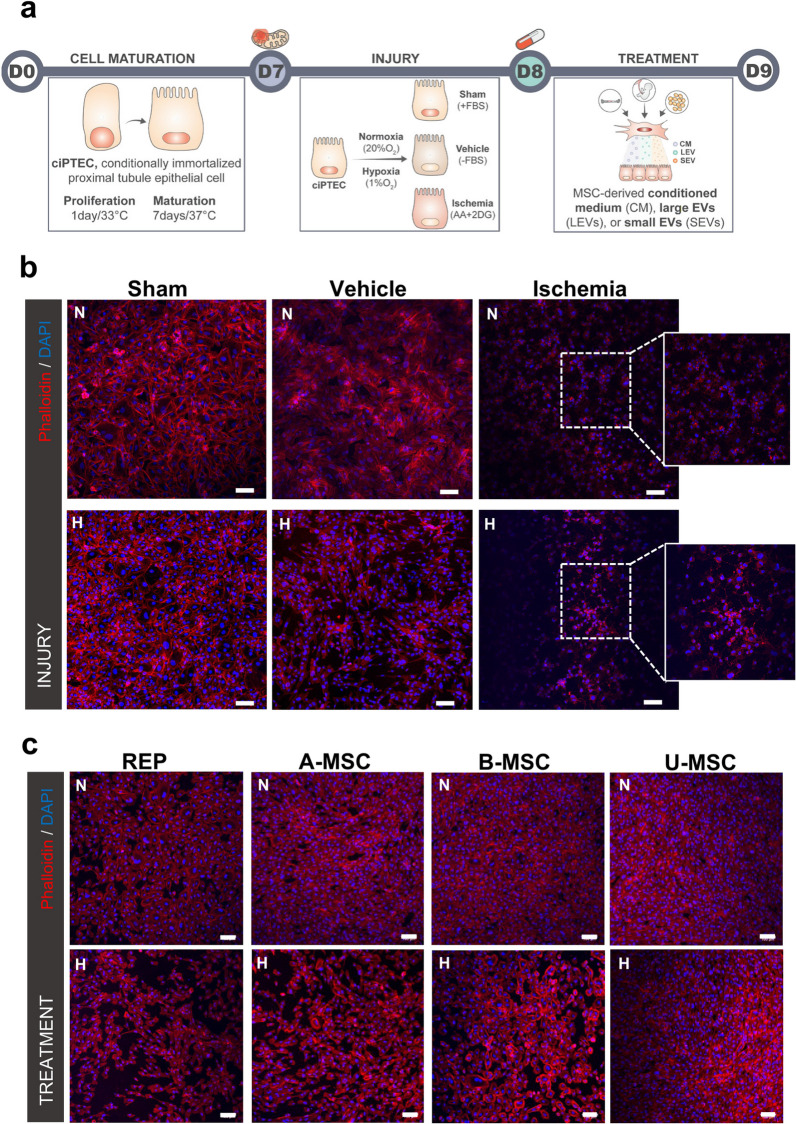
Morphological rearrangements following in vitro ischemia and MSC treatment. **a** Schematic overview of the protocol for inducing ischemia-like effects in human ciPTEC line. Sham: ciPTECs cultured in serum-containing medium; Vehicle: ciPTECs cultured in serum-free medium; Ischemia: ciPTECs cultured in serum-free medium containing antimycin AA (AA) and 2-deoxy-D-glucose (2DG) **b** Immunofluorescence of the cellular organization of ciPTECs cultured under normoxia (N) and hypoxia (H) conditions showed cytoskeleton derangements. **c** MSC-derived secretome partially reverted alterations in cell morphology. Representative images obtained using confocal microscopy showing: in blue: DAPI (nuclei staining), in red: Phalloidin (binds to F-actin filaments). Scale bar: 100 µm. *REP* reperfusion, *A-/B-/U-MSC* adipose-/bone marrow-/umbilical cord-derived mesenchymal stromal cells, *N* normoxia, *H* hypoxia

To model ischemia in vitro, cells were treated with antimycin A (AA) and 2-deoxyglucose (2DG) to inhibit mitochondrial respiration and glycolysis, respectively, under normoxia (N-ciPTECs) and hypoxia (H-ciPTECs) conditions (Fig. 1a). N-ciPTECs were used as control conditions of the oxygen-deprived group. This injury treatment led to disruption of the F-actin network and a decrease in the number of nuclei per surface area, particularly in the hypoxic group (Fig. 1b). H-ciPTECs increased collagen IV deposition when co-treated with TGF-β, while this was not observed in the sham, vehicle and ischemic groups, confirming the efficacy of our model to mimic the progression to chronic kidney disease observed after AKI (Additional file 1: Fig. S1). Treatment with MSC-secretome improved cell morphology in a qualitative analysis in N-ciPTECs as evidenced by cytoskeleton staining, regardless of the source of MSCs (Fig. 1c). However, MSC therapy was less effective in H-ciPTECs, as observed by the presence of holes in the monolayers (Fig. 1c).

### Treatment with MSCs secretome ameliorates mitochondrial dysfunction in experimental ischemia

**Fig. 2:**
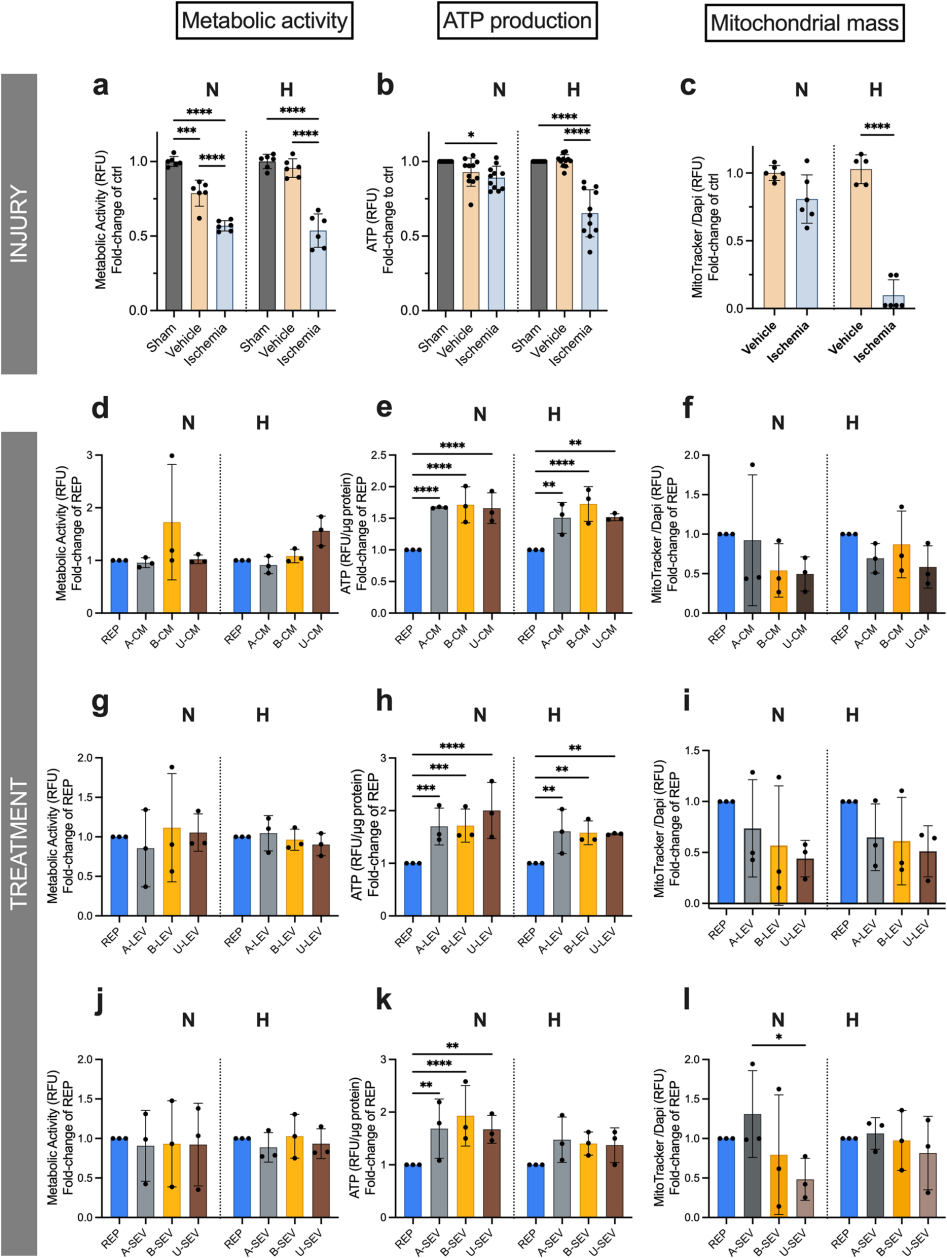
In vitro characterization of metabolic and mitochondrial amelioration following MSC therapy. To evaluate the level of metabolic dysfunction after ischemic injury, we looked into metabolic activity (**a**), ATP production (**b**), and mitochondrial mass (**c**). Similarly, effects of the treatment with CM (**d–f**), LEV (**g–i**), and SEV (**j–l**). Data are shown as mean ± SD of three replicates from three independent experiments. One-way ANOVA statistical analysis performed (*p value < 0.05; **p value < 0.01; ***p value < 0.001; ****p value < 0.0001)

**Fig. 3:**
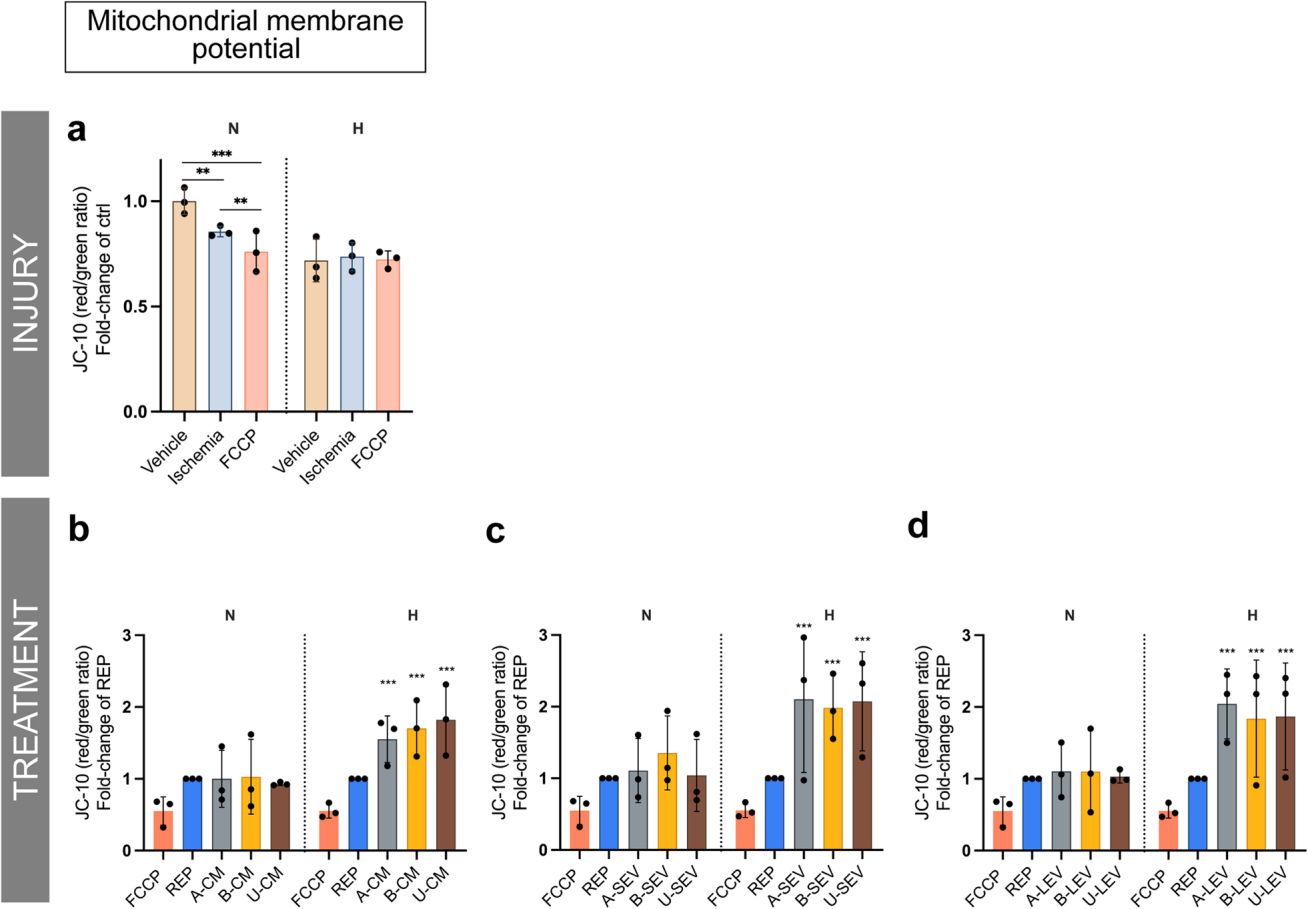
In vitro characterization mitochondrial membrane potential following MSC therapy. To evaluate the level of mitochondrial dysfunction after ischemic injury we looked into mitochondrial membrane potential levels following (**a**) injury. Similarly, effects of the treatment with CM (**b**), SEV (**c**), and LEV (**d**). Data are shown as mean ± SD of three replicates from three independent experiments. FCCP was used as a positive control for depolarized mitochondrion. One-way ANOVA statistical analysis performed (*p value < 0.05; **p value < 0.01; ***p value < 0.001; ****p value < 0.0001)

During cellular ischemia and/or hypoxia, the cells experience metabolic and mitochondrial dysfunctions due to lack of nutrients and oxygen supply. In serum-deprived (vehicle) ciPTECs, there was a decrease in metabolic activity (Fig. 2a) and in ATP production (Fig. 2b), which was more pronounced in the ischemia group and under hypoxia. These changes were consistent with a reduction in mitochondrial mass (Fig. 2c) and a decreased mitochondrial membrane potential (Δψ) (Fig. 3a). ROS production increased gradually over time in N-ciPTECs (Additional file 2: Fig. S2a), while in H-ciPTECs ROS production increased but to a lesser extent (Additional file 2: Fig. S2b), suggesting that the combination of chemically induced ischemia and hypoxia may interfere with ROS generation.

During experimental reperfusion, B-CM treatment slightly increased the metabolic activity in ischemic N-ciPTECs and U-CM was somewhat effective in H-ciPTECs (Fig. 2d). The remaining treatments showed similar levels (Fig. 2g–j), indicating that MSC therapy can (partially) restore cellular metabolic activity. All MSC-derived treatments (CM, LEV, SEV) significantly increased ATP production in N-ciPTECs (Fig. 2e, h, k), with U-LEV (Fig. 2h) and B-SEV (Fig. 2k) having stronger effects. Similarly, CM and LEVs treatment from all sources significantly increased ATP in H-ciPTECs (Fig. 2e, h). Interestingly, CM and LEV treatment reduced mitochondrial mass (Fig. 2f, i), whereas SEV treatment, regardless of the source, revealed similar mitochondrial mass levels in H-ciPTECs (Fig. 2l). This suggests that the effects of different MSC-derived therapies on mitochondrial mass may vary. In N-ciPTECs, MSC treatment showed comparable levels of Δψ (Fig. 3a). Distinctively, all MSC-derived treatments enhanced Δψ in H-ciPTECs compared with the no treatment control (Fig. 3b–d).

Overall, MSC-derived secretome can restore metabolic activity and increase ATP levels, critical for cell survival and injury resolution following reperfusion.

## MSC therapy rescues energetic phenotype by increasing glycolytic rates in ischemic kidney proximal tubule cells

**Fig. 4:**
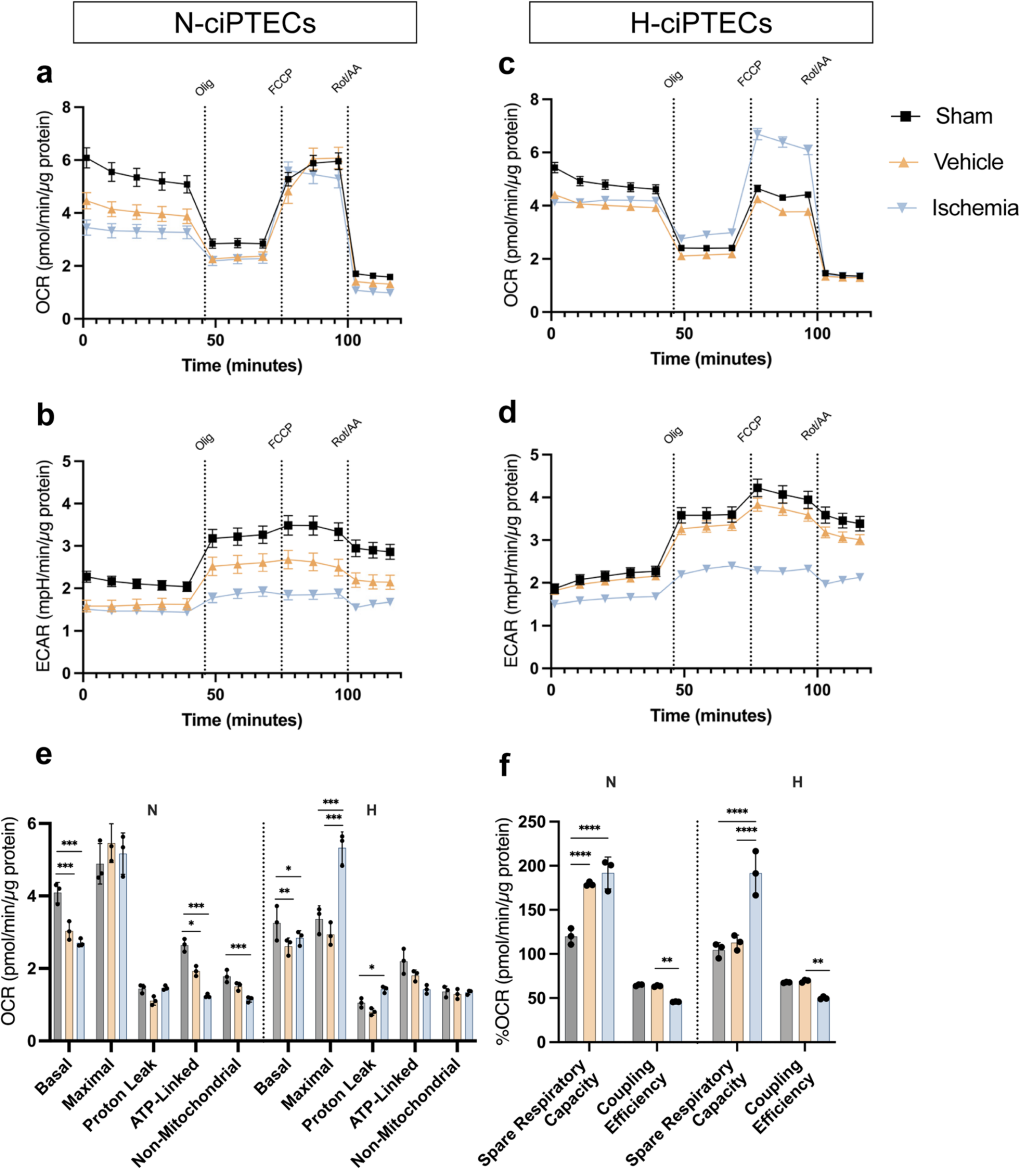
Bioenergetic profiles of ciPTECs after ischemia. **A, C** OCR and **B, D** and ECAR levels measured before and after injections of oligomycin, FCCP, and rotenone/antimycin A. Specific parameters derived from OCR levels from N- and H-ciPTECs (**E, F**) were plotted in bar graphs. Data are shown as mean ± SD of six replicates from three independent experiments. One-way ANOVA statistical analysis performed (*p value < 0.05; **p value < 0.01; ***p value < 0.001; ****p value < 0.0001)

Under ischemia, metabolic dysfunction is induced by alterations in the mitochondrial ETC resulting in diminished metabolic activity and ATP production. The bioenergetic status of the cells was affected, as evidence by reduced oxygen consumption rate (OCR) (Fig. 4a, b) and extracellular acidification rate (ECAR) (Fig. 4c, d) in both vehicle and ischemia groups. Upon starvation and particularly during ischemia, cells showed a decline in their energetic space dimensions, which restricted metabolism that hampers cell survival and function (Additional file 3: Fig. S3a, b). Similarly, basal and ATP-linked respiration were reduced in the vehicle and ischemia groups (Fig. 4e), reflecting a decline in the cellular capacity to produce ATP via oxidative phosphorylation (OXPHOS). Hypoxic conditioning led to a more severe damage to the inner mitochondrial membranes (IMM) and/or the ETC, as demonstrated by a decline in coupling efficiency and an increase in proton leak (Fig. 4e). The spare respiratory capacity (SRC) is an important measure of cellular ability to respond to stress by increasing energy demand. We found that SRC was significantly increased in both vehicle and ischemic N-ciPTECs (Fig. 4f). In H-ciPTECs, this effect was less prominent and occurred only in the vehicle group (Fig. 4f).

**Fig. 5:**
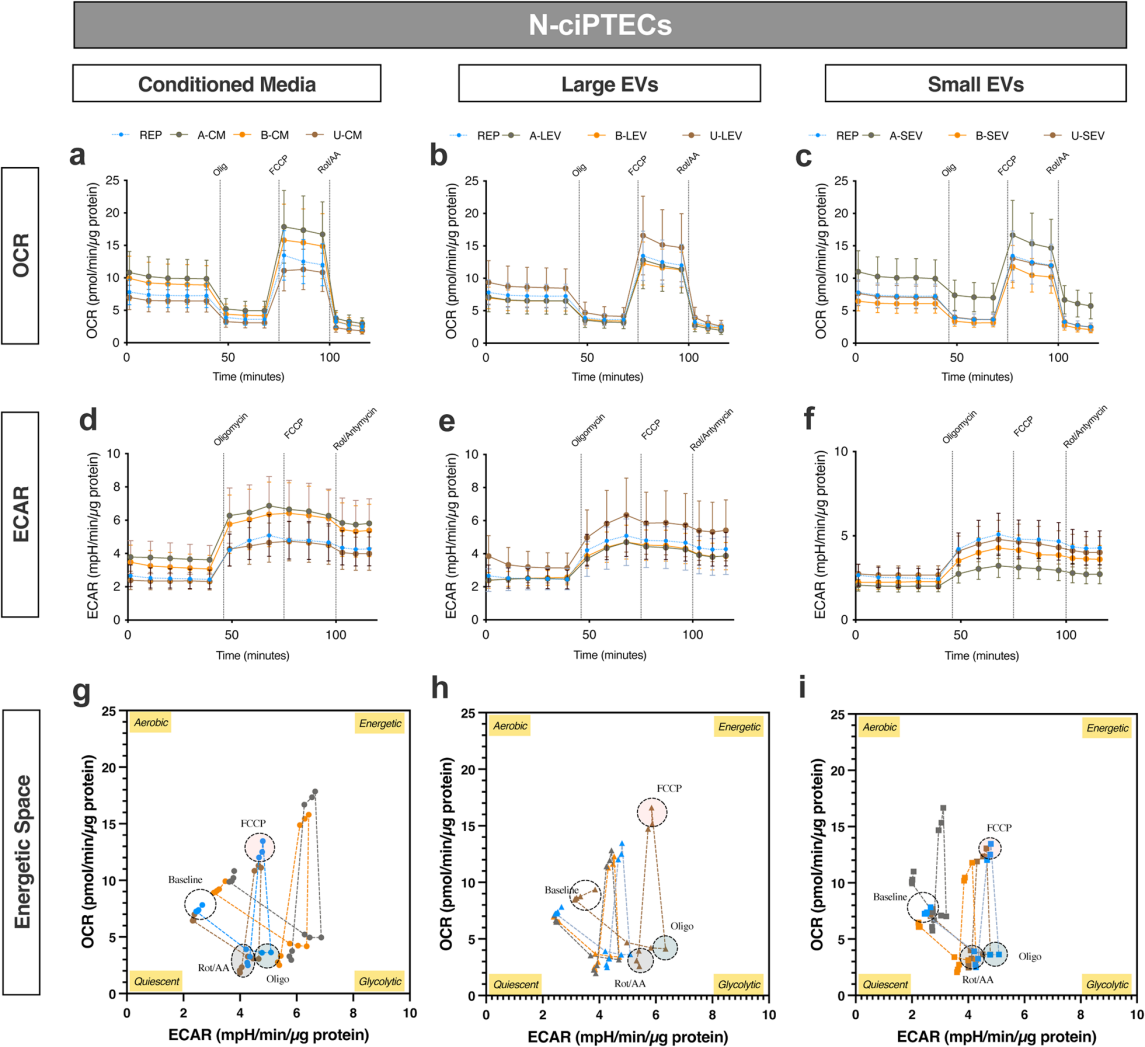
Bioenergetic profile of ischemic N-ciPTECs upon MSC therapy. OCR (**a–c**) and ECAR (**d, e**) levels were measured before and after injections of oligomycin, FCCP, and rotenone/antimycin A. Energetic space of MSC-treated ischemic ciPTECs (**g–i**). Data are shown as mean ± SD of 6 replicates of three independent experiments

**Fig. 6:**
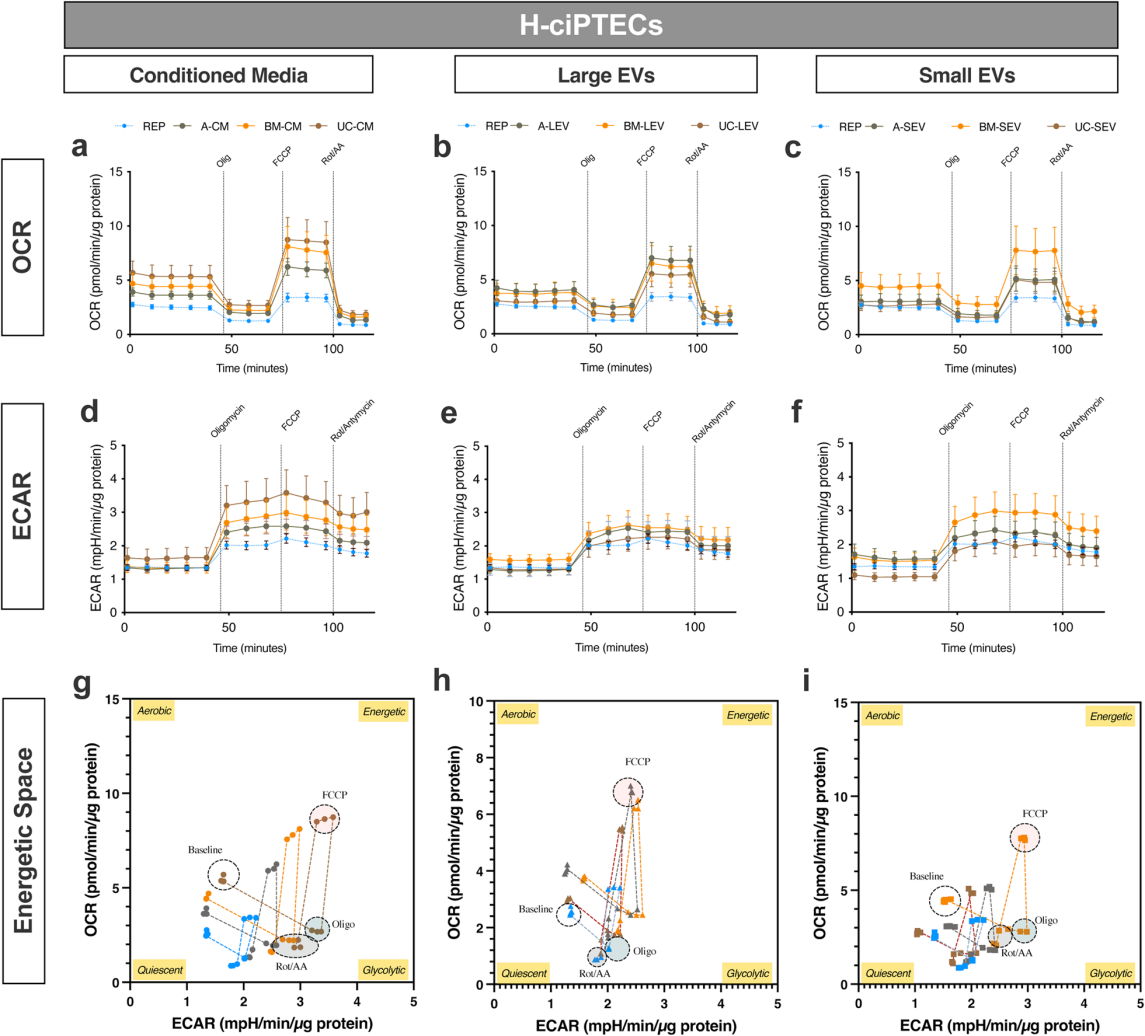
Bioenergetic profile of ischemic H-ciPTECs upon MSC therapy. OCR (**a–c**) and ECAR (**d, e**) levels were measured before and after injections of oligomycin, FCCP, and rotenone/antimycin A. Energetic space of MSC-treated ischemic ciPTECs (**g–i**). Data are shown as mean ± SD of 6 replicates of three independent experiments

Treatment with MSC secretome rescued the bioenergetic status of N-ciPTECs (Fig. 5) to a greater extent than in H-ciPTECs (Fig. 6). Specifically, in normoxia, A-CM and B-CM together with U-LEVs and A-SEVs yielded higher OCR and ECAR values than the reperfusion group (Fig. 5a–f). Furthermore, A- and B-CM showed an increase in the energetic space (Fig. 5g). U-LEVs generated a similar bioenergetic profile (Fig. 5h), while MSC-SEVs generated a more restricted space (Fig. 5i). The OCR-related parameters in CM-treated cells increased in basal and maximal respiration (Additional file 4: Fig S4a). Additionally, LEVs, and especially B-LEVs, increased the maximal respiration levels compared to the reperfusion treatment (Additional file 4: Fig S4b), while B-SEV showed no effect (Additional file 4: Fig S4c). All treatments, except for A-MSCs, showed a tendency to increase SRC and maintain similar levels of coupling efficiency, indicating a recovery in the ETC and/or IMM health status (Additional file 4: Fig S4d–f). In H-ciPTECs, all MSC treatments induced bioenergetics of oxygen consumption compared to the reperfusion group (Fig. 6a–c), but only CM from all sources and B-SEVs increased the ECAR profile (Fig. 6d–f). The energetic space of CM-treated ciPTECs occupied a larger space when compared to the reperfusion group, indicating the induction of an energetic phenotype (Fig. 6g–i). Specifically, B- and U-CM treatments showed a tendency to increase basal levels of oxygen consumption and increased the maximal respiration levels achieved (Additional file 4: Fig S4a), similar to what was observed for B-SEVs (Additional file 4: Fig S4c). All treatments slightly increased SRC and lowered coupling efficiency rates (Additional file 4: Fig S4d–f). The vehicle and ischemia groups showed an increase in their glycolytic profile (*J*_ATPglyc_) relative to the sham group (Additional file 5: Fig. S5a, b), likely due to compensation for OXPHOS inhibition. Both in normoxia and hypoxia, the ischemia group exhibited a decrease in *J*_ATPox coupled_ without changes in *J*_ATPox TCA_ (Additional file 5: Fig. S5a, b). Similarly, glycolysis remained to be the primary source of ATP following MSC treatment (Additional file 5: Fig. S5c–h). In N-ciPTECs, both B- and U-EVs showed similar trends (Additional file 5: Fig. S5d, e), while A-EVs induced distinct responses: A-LEV exhibited a trend of increased *J*_ATPox coupled_, whereas A-SEV increased *J*_ATPglyc_ compared to the reperfusion group (Additional file 5: Fig. S5e). In contrast, in H-ciPTECs, CM treatment reduced *J*_ATPglyc_ (Additional file 5: Fig. S5f), while B- and U-treatments increased *J*_ATPox coupled_ (Additional file 5: Fig. S5f–h). Further, LEVs showed similar percentages to the reperfusion group (Additional file 5: Fig. S5g) and only B-SEVs increased *J*_ATPox coupled_ (Additional file 5: Fig. S5h).

## MSC therapy increases glycolytic activity of ischemic ciPTECs and reduces oxidative stress after chemically induced ischemia

**Fig. 7:**
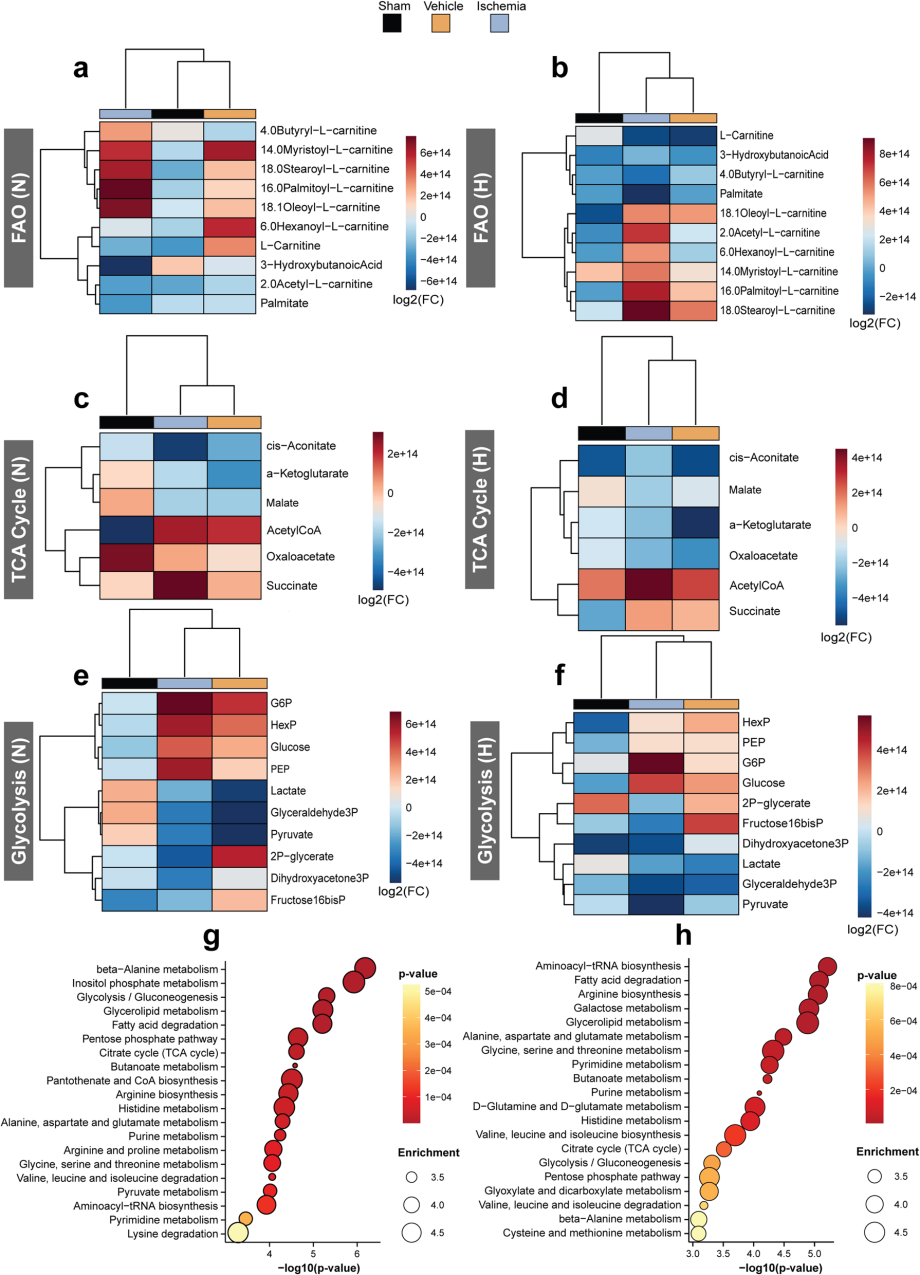
Metabolomics profile of ischemic ciPTECs in normoxia and hypoxia. Heatmaps of the key metabolites involved in fatty acid oxidation (FAO) (**a, b**), tricarboxylic acid (TCA) cycle (**c, d**), and glycolysis (**e, f**). Metabolite sets enrichment overview of ischemic N- ciPTECs (**g**) and H-ciPTECs (**h**), compared to sham, representing their physiological relevance. Heatmap results are shown as log_2_(FC) compared to the sham group. Red colors indicate increased values, while blue colors indicate decreased values. N, normoxia; H, hypoxia

After ischemic injury, changes in fatty acid metabolism were characterized by an increased accumulation of long-chain acylcarnitines (LCAC), compounds required for the transfer of fatty acids in and out of mitochondria, whereas for the sham group, the opposite was observed (Fig. 7a, b). These findings can point to an altered catabolic capacity, since fatty acids can be used to fuel the TCA cycle via β-oxidation. In addition, depletion of TCA cycle intermediates is in line with lower OXPHOS, indicating lower mitochondrial energy production with the exception of succinate (Fig. 7c, d), which favors the development of a microenvironment for oxidative stress and apoptosis [44].

Ischemic cells had higher levels of upstream glycolytic intermediates, such as glucose 6-phosphate (G6P) and other hexose-phosphates (HexP), and phosphoenolpyruvate (PEP), which can be converted into pyruvate. Reduced downstream glycolytic metabolites in ischemic groups indicated the successful inhibition of glucose metabolism by 2DG (Fig. 7e, f). Together with the reduced levels of TCA cycle intermediates, this confirms a lower energy metabolism. A metabolite set enrichment analysis was performed to investigate the molecular pathways altered in ischemic ciPTECs. Results showed that in N-ciPTECs (Fig. 7g), the enriched metabolic pathways were beta-alanine metabolism, inositol phosphate metabolism, glycerolipid metabolism and fatty acid degradation, suggesting a priority for energy production and protection against cellular damage. In H-ciPTECs (Fig. 7h), the enriched metabolic pathways were fatty acid degradation, glycerolipid metabolism, glycine, serine and threonine metabolism, with a priority for energy production and essential biomolecule synthesis.

**Fig. 8:**
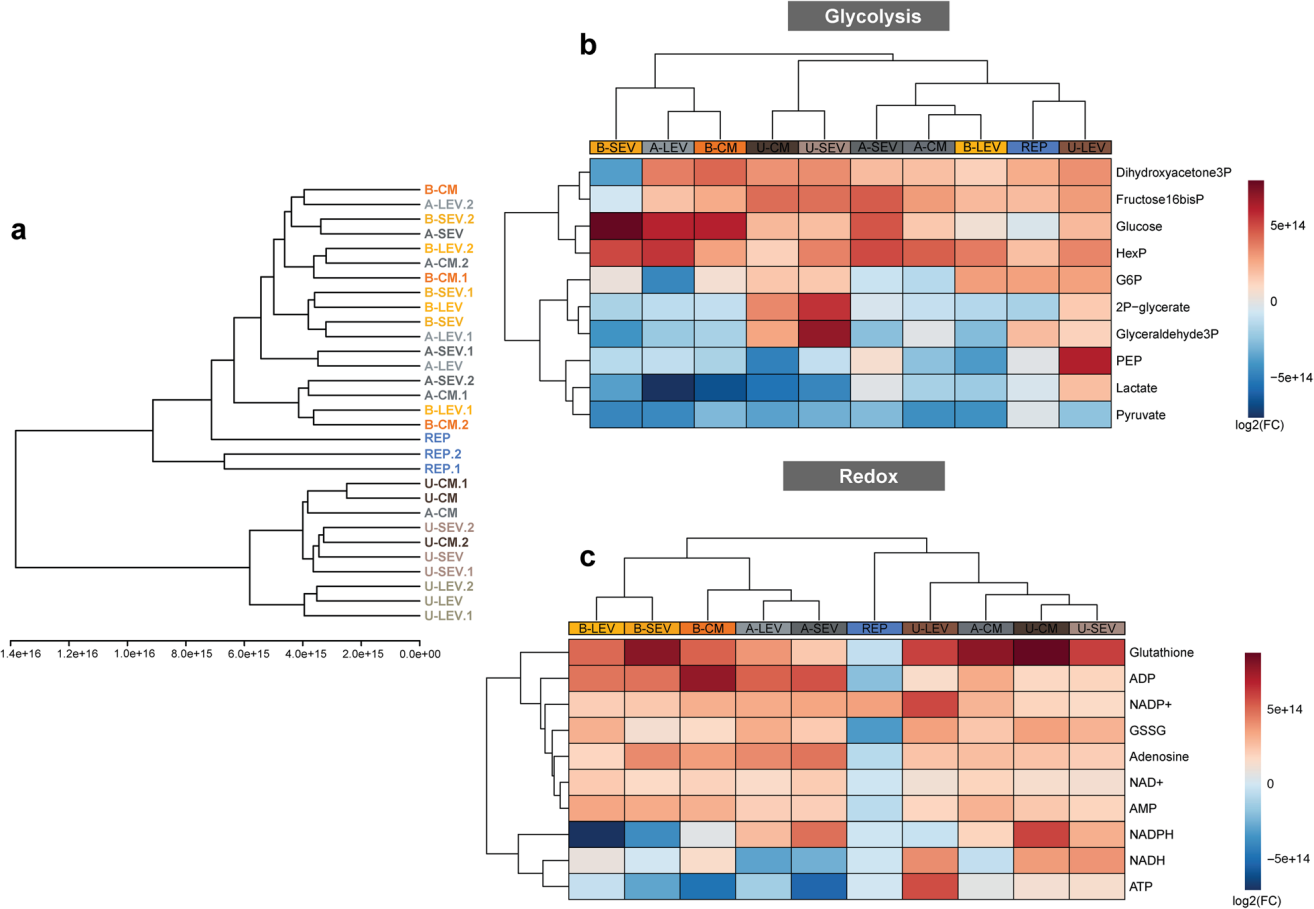
Metabolomics profile of normoxia-treated ischemic ciPTECs (N-ciPTECs). **a** Hierarchical cluster analysis of the different conditions tested. **b** Heatmaps of the key metabolites involved in glycolysis (**c**) and redox. *A* adipose tissue, *B* bone marrow, *U* umbilical cord, *CM* conditioned medium, *LEV* large EVs, *SEV* small Evs. Data are shown as log_2_ fold-change of the reperfusion group; Red colors indicate increased values, while blue colors indicate decreased values

**Fig. 9:**
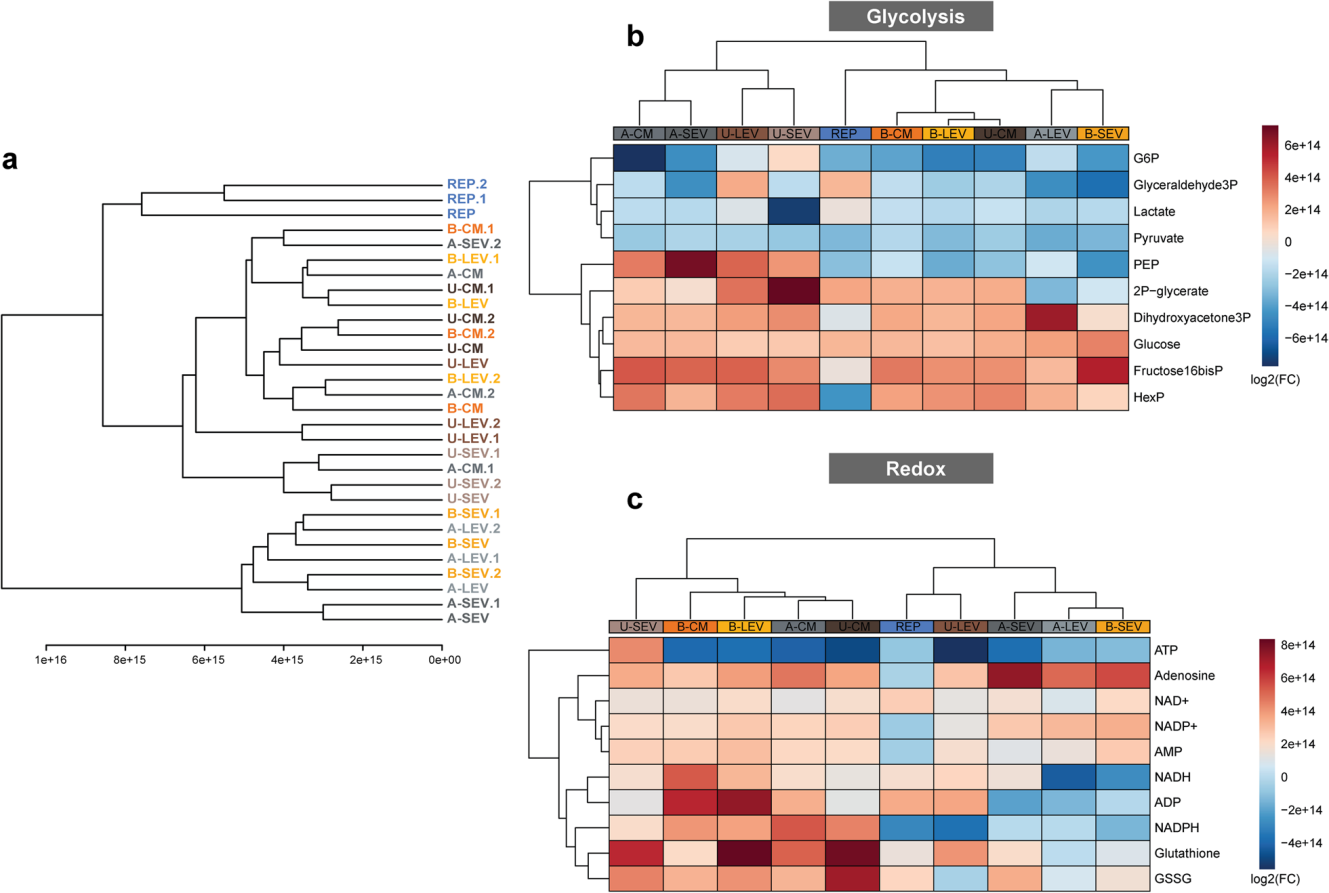
Metabolomics profile of hypoxia-treated ischemic ciPTECs (H-ciPTECs). **a** Hierarchical cluster analysis of the different conditions tested. **b** Heatmaps of the key metabolites involved in glycolysis (**c**) and redox. *A* adipose tissue, *B* bone marrow, *U* umbilical cord, *CM* conditioned medium, *LEV* large-sized extracellular vesicles, *SEV* small-sized extracellular vesicles. Data are shown as log_2_ fold-change of the reperfusion group; Red colors indicate increased values, while blue colors indicate decreased values

Metabolomic analysis following experimental reperfusion indicated that A- and B-MSC secretome in ischemic N-ciPTECs elicited similar metabolic effects as the positive control. In contrast, U-MSC secretome clustered distinctly (Fig. 8a). In ischemic H-ciPTECs, the reperfusion showed an heterogenous metabolic profile as multiple treatments were clustered together, while B-SEV, A-LEV and A-SEV clustered the furthest (Fig. 9a). Considering the improvements observed in ATP content and the predominantly glycolytic phenotype of the cells, we investigated the metabolic changes associated with these pathways. In ischemic N-ciPTECs, U-MSC secretome, particularly U-LEV, showed the most prominent improvements in cellular bioenergetics, as evidenced by increased levels of glycolysis intermediates (Fig. 8b), NADH and NADPH (Fig. 8c), as well as a reduction in oxidized metabolites such as NAD+ and NADP+ (Fig. 8c). Additionally, U-MSC secretome showed an increase in reduced and oxidated glutathione levels (Fig. 8c), suggesting improved redox balance. In ischemic H-ciPTECs, U-SEV and U-LEV showed increased levels of glycolytic intermediates (Fig. 9b), indicating an upregulation of glycolysis, with U-SEV showing the highest increase in ATP (Fig. 9c). On the other hand, B-CM, B-LEV, and U-CM treatments resulted in an increase in NADH, NADPH, GSSG, and glutathione (Fig. 9c), indicating an accumulation of reducing equivalents, at a possible lower rate of consumption leading to a decrease in ATP (Fig. 9c). Moreover, the metabolite set enrichment analysis demonstrated an enrichment of pentose phosphate pathway in either N-ciPTECs and H-ciPTECs (Additional file 6: Fig. S6), which supports a potential antioxidant therapeutic effect.

## Discussion

Using an optimized in vitro model, we here demonstrate that the MSC secretome reduces oxidative stress and improves energy production following ischemic injury. We evaluated three different sources of MSC secretome and demonstrated that critical hallmarks of the acute ischemic phase, including cytoskeletal rearrangements, energetic imbalance, increased oxidative stress, mitochondrial dysfunction, and altered antioxidant defenses and redox homeostasis, were achieved at 24 h following ischemic induction and were enhanced further under hypoxia. During the reperfusion phase, viz*.* treatment with MSC secretome, particularly U-MSC restored cell morphology and metabolic derangements, partially reversing ischemia-induced damage. The described improvements in cell morphology could be derived from changes in the activation of cell death programs that typically convey during severe ischemic damage [45], as presented by others [46–48], and should be explored further in follow-up studies. To our knowledge, this is the first in vitro study to compare the therapeutic efficacy of three sources of MSC secretome in this setting.

Proximal tubule cells are highly susceptible to ischemic injury, and their metabolic and mitochondrial function are critical to maintaining renal homeostasis [49]. A decline in mitochondrial activity leads to a reduction in ATP production and an increase in the release of harmful products, such as ROS as seen in injured cells. Following ischemia, a loss of mitochondrial content and impaired mitochondrial activity was reflected in the bioenergetic profile, with decreased oxygen consumption. Morphological and functional alterations in the mitochondrial pool following an ischemic insult may also impact the recovery phase [50]. The improved bioenergetic status, despite reduced mitochondrial mass, following MSC treatment may reflect the clearance of damaged mitochondria and the initiation of biogenesis and regeneration. In agreement, U-MSC EVs were found to alleviate mitochondrial fragmentation [51] and B-MSCs and their SEVs promoted mitophagy through microRNA-dependent mechanisms [52], supporting their renoprotective potential in favor of tissue regeneration.

The metabolomic profile of ischemic cells demonstrated imbalanced fatty acid metabolism and a general defect in mitochondria’s catabolic activity as seen by the accumulation of metabolic byproducts, such as acylcarnitines that contribute to cellular damage and apoptosis [53, 54]. The TCA cycle activity is dependent on the availability of oxygen and nutrients, thus imbalanced FAO may alter the TCA cycle activity. The accumulation of acetyl-coA, an intermediate in both pathways, is a hallmark of this dysfunction due to its effects on inhibiting enzymes involved in the TCA cycle, exacerbating energy deficit [55, 56]. The metabolic rewiring that takes place during acute injury aimed to sustain cell viability is characterized by an increase in glycolytic rates [57–60], an effect widely described in the literature that supports the increase seen in key upstream glycolytic metabolites. Other downstream effects, such as glucose 6-phosphate or PEP, were found decreased following ischemia. PEP has been shown to exert cytoprotective and anti-oxidative properties that prevent a decrease in ATP content [61, 62], which could explain the moderate decrease in ATP under normoxic culture. While an increase in ATP production was observed across all treatments following ischemia, the metabolic profile of treated ischemic cells varied. In N-ciPTECs, larger changes in bioenergetics and redox homeostasis were mainly exerted by U-MSC therapy. Similar results were seen in H-ciPTECs, despite their metabolic profiles being more heterogeneous and higher oxidative imbalance. The changes found are in line with previous findings that suggest U-MSCs mediate the recovery process following IRI through various mechanisms that lead to reduced oxidative stress [8, 63] and increased energy balance [64], critical to ensure cell viability following ischemic insults.

All U-MSC secretome, and in particular U-LEV, showed an enrichment in PPP and beta-alanine, with N-ciPTECs exhibiting a greater enrichment compared to H-ciPTECs. Increased NAD metabolism has been identified as a protection mechanism by restoring oxidative metabolism [65], stimulating mitochondrial biogenesis and increasing oxygen consumption [66, 67]. In addition, PPP not only generates NADPH but also ribulose-5-phosphate, which is essential for nucleotide synthesis as well as ATP synthesis. Additionally, beta-alanine might indirectly support ATP production by supporting the function of enzymes involved in ATP synthesis [68]. This increase in bioenergetics and redox homeostasis is further supported by previous studies that have shown the presence of mitochondrial components and antioxidant miRNAs in EVs, providing metabolic support to damaged cells [69–71].

We found that for each MSC source, the respective LEVs and SEVs showed similar metabolic profiles in the treated cells. However, the mechanism behind their therapeutic efficacy was not clear because they had similar basal respiration, ATP production, SRC and nonmitochondrial respiration levels. We found that A- and B-EV treated N-ciPTECs resulted in a significant increase in SRC compared to the reperfusion group, which has been shown to be regulated by AMPK [72, 73]. This increase in SRC has been demonstrated in cases of metabolic stress, in which cells show increased cell survival by adapting to oxidative stress. The extrapolated metabolite enrichment pathway analysis of U-MSC-treated N- and H-ciPTECs showed a difference in pathways. One possible explanation for these results is that the metabolic response to ischemia differs depending on the severity of the injury, with H-ciPTECs being subjected to a more energy deficient microenvironment, hence the enrichment in the TCA cycle and glycerolipid metabolism. We suggest that the superior therapeutic effect of U-MSC may be due to their embryonic origin, which is thought to confer higher survival rate, and most importantly, a richer secretome [74, 75]. In addition to increasing ATP content, MSC therapy partially reverted the disruption of the actin cytoskeleton observed in ischemic ciPTECs. While this effect was attributed to an increase in ATP, a key player in the polymerization of actin filaments [76], heat shock proteins (HSPs) has also been shown to improve the repair of structural proteins after ischemia-induced cytoskeleton damage [77]. In an earlier study, HSPs have been identified in a proteomic analysis of MSCs, suggesting that they may contribute to the therapeutic effect of MSC secretome [78].

While our in vitro model effectively reproduces molecular and morphological changes seen in vivo during renal IRI and offers valuable insights into the therapeutic effect of the MSC secretome, it has inherent limitations. These include the use of a single cell type in a two-dimensional monolayer, which incompletely represents the adult kidney. The incorporation of additional cell types will improve the physiological relevance of our model and give an in-depth understanding of the therapeutic effect of MSC secretome. For instance, T-cells, particularly CD3^+^ T-cells, have been shown to play a significant role in mediating post-ischemic damage by increasing the release of pro-inflammatory cytokines [79], whereas regulatory T-cells have an anti-inflammatory role in IRI [80]. Furthermore, microvascular leakage is another hallmark of IRI, exacerbating ischemic damage by inducing, among others, the release of inflammatory cytokines by leukocytes (e.g., lymphocytes) [81, 82]. To address this, integrating models like organ-on-chip systems would provide a more accurate representation of the complex cellular interplay between the various cell types. An example of such a model was developed in which PTECs and endothelial cells were exposed to hypoxia and normoxia to recreate the IRI microenvironment, and the therapeutic efficacy of vitamin therapy in ameliorating the damage could be demonstrated [83]. Traditionally, IRI and therapies for ischemic damage are mainly studied and tested in rodent models, but the variability of the injury and response to therapy between individual animals makes it difficult to obtain consistent results. Additionally, in vivo models may not fully replicate the complex cellular and molecular interactions that occur during IRI in humans as well, which limits their translational value. Therefore, human-based in vitro models, such as organ-on-chip, could potentially be valuable for studying the cellular and molecular mechanisms underlying biological processes in health and disease and for developing and testing new therapeutic strategies.

## Conclusions

Altogether, we showcase the advantage of using a chemically induced ischemia model in combination with hypoxia to more accurately mimic the physiological cellular response to ischemia and to evaluate the benefits of MSC-derived therapies during experimental reperfusion. Further research is needed to determine the exact mechanism of action of MSCs, in particular the effects seen by U-MSC secretome. Additionally, dose–response studies should be taken along to evaluate optimal therapeutic delivery. Nonetheless, our data expand the current understanding that the PT is highly susceptible to ischemic damage and showcases the therapeutic effect of MSC secretome in improving the bioenergetic profile of ischemic PT cells.

### Supplementary Information

**Additional file 1**. **Figure S1.** Immunofluorescence of the cellular organization of ciPTECs cultured under normoxia (**N**) and hypoxia (**H**) conditions. TGF-β (pro-fibrotic mediator) was used as positive control. In blue: DAPI (nuclei staining), in green: collagen IV. Scale bar: 100 mm.

**Additional file 2**. **Figure S2.** (**a-b**) Intracellular reactive oxygen species (ROS). Data are shown as mean ± SD of four replicates of three independent experiments. H_2_O_2_ was used as a positive control. Two-way ANOVA statistical analysis performed with Tukey’s multiple comparisons test (*p value < 0.05; **p value < 0.01; ***p value < 0.001; ****p value < 0.0001). N, normoxia; H, hypoxia.

**Additional file 3**. **Figure S3.** Bioenergetic alterations following ischemic conditioning in kidney proximal tubule cells. (**a**, **b**) Energetic space generated by plotting OCR vs ECAR levels before and after injections of oligomycin, FCCP, and rotenone/antimycin A. Data are shown as mean ± SD of ten replicates of three independent experiments. One-way ANOVA statistical analysis performed (*p value < 0.05; **p value < 0.01; ***p value < 0.001; ****p value < 0.0001). N, normoxia; H, Hypoxia.

**Additional file 4**. **Figure S4.** Breakdown of OCR-related parameters of MSC-treated N-ciPTECs and H-ciPTECs of CM (**a**,**d**), Large EVs (**b**,**e**), and Small EVs (**c**,**f**). Data are shown as mean ± SD of 6 replicates of three independent experiments. Statistical analysis performed using Two-Way ANOVA with Dunnett’s post-hoc test. *p value < 0.05; **p value < 0.01; ***p value < 0.001; ****p value < 0.0001). N, normoxia; H, hypoxia.

**Additional file 5**. **Figure S5.** Net rate of ATP production (J_ATP_) of ischemic ciPTECs (**a**,**b**), and ischemic ciPTECs treated with MSC secretome (**c**-**h**). The J_ATP_ divided in its three main components (J_ATPglyc_, J_ATPox coupled_, J_ATPox TCA_). Statistical analysis performed using Two-way ANOVA and Dunnet’s post-hoc test (*p value < 0.05). Data are shown as mean ± SD of 6 replicates of three independent experiments.

**Additional file 6**. **Figure S6.** Metabolite sets enrichment overview showing the most altered metabolites revealed in ischemic ciPTECs treated with U-CM (**a**,**b**), U-LEV (**c**,**d**), or U-SEV (**e**–**f**), representing their physiological relevance. The top 25 metabolites for each treatment were compared to the reperfusion group (positive control). N, normoxia; H, hypoxia.

**Additional file 7**. Immunofluorescence analysis antibodies.

## Data Availability

Data from the current study are available from the corresponding author on reasonable request. Metabolomics data are deposited at MetaboLights with identifier MTBLS6279.

## Abbreviations

2DG: 2-Deoxy-d-glucose
A: Adipose tissue
AA: Antimycin A
AKI: Acute kidney injury
B: Bone marrow
ciPTECs: Conditionally immortalized proximal tubule epithelial cells
CM: Conditioned medium
ECAR: Extracellular acidification rate
ETC: Electron transport chain
EVs: Extracellular vesicles
FAO: Fatty acid oxidation
H: Hypoxia
IMM: Inner mitochondrial membrane
IRI: Ischemia/reperfusion injury
LEVs: Large extracellular vesicles
MSC: Mesenchymal stromal cell
N: Normoxia
OCR: Oxygen consumption rate
OXPHOS: Oxidative phosphorylation
PTEC: Proximal tubule epithelial cell
ROS: Reactive oxygen species
SEVs: Small extracellular vesicles
SRC: Spare respiratory capacity
U: Umbilical cord
