# Supplementary figures and images for: Mesenchymal stromal cells secretome restores bioenergetic and redox homeostasis in human proximal tubule cells after ischemic injury
Source: Stem Cell Res Ther. 2023 Dec 10;14:353. doi: 10.1186/s13287-023-03563-6 (PMC10712181; doi:10.1186/s13287-023-03563-6)

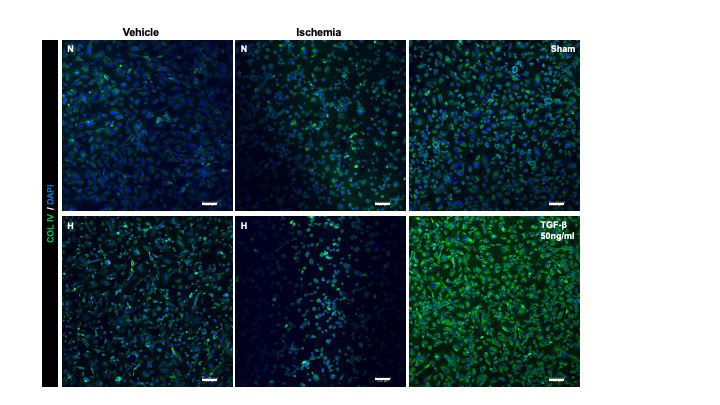

Supplement: Supplementary file 1 — Additional file 1. Figure S1. Immunofluorescence of the cellular organization of ciPTECs cultured under normoxia (N) and hypoxia (H) conditions. TGF-β (pro-fibrotic mediator) was used as positive control. In blue: DAPI (nuclei staining), in green: collagen IV. Scale bar: 100 mm. [file 13287_2023_3563_MOESM1_ESM.tiff]

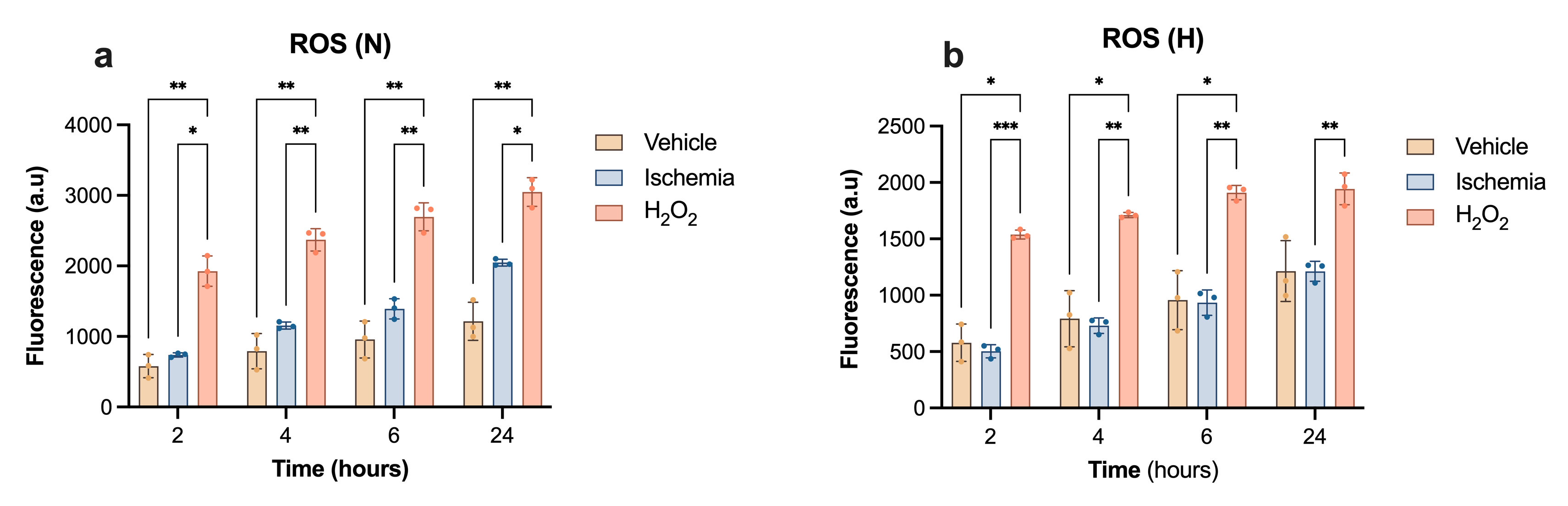

Supplement: Supplementary file 2 — Additional file 2. Figure S2. (a-b) Intracellular reactive oxygen species (ROS). Data are shown as mean ± SD of four replicates of three independent experiments. H2O2 was used as a positive control. Two-way ANOVA statistical analysis performed with Tukey’s multiple comparisons test (*p value < 0.05; **p value < 0.01; ***p value < 0.001; ****p value < 0.0001). N, normoxia; H, hypoxia. [file 13287_2023_3563_MOESM2_ESM.tiff]

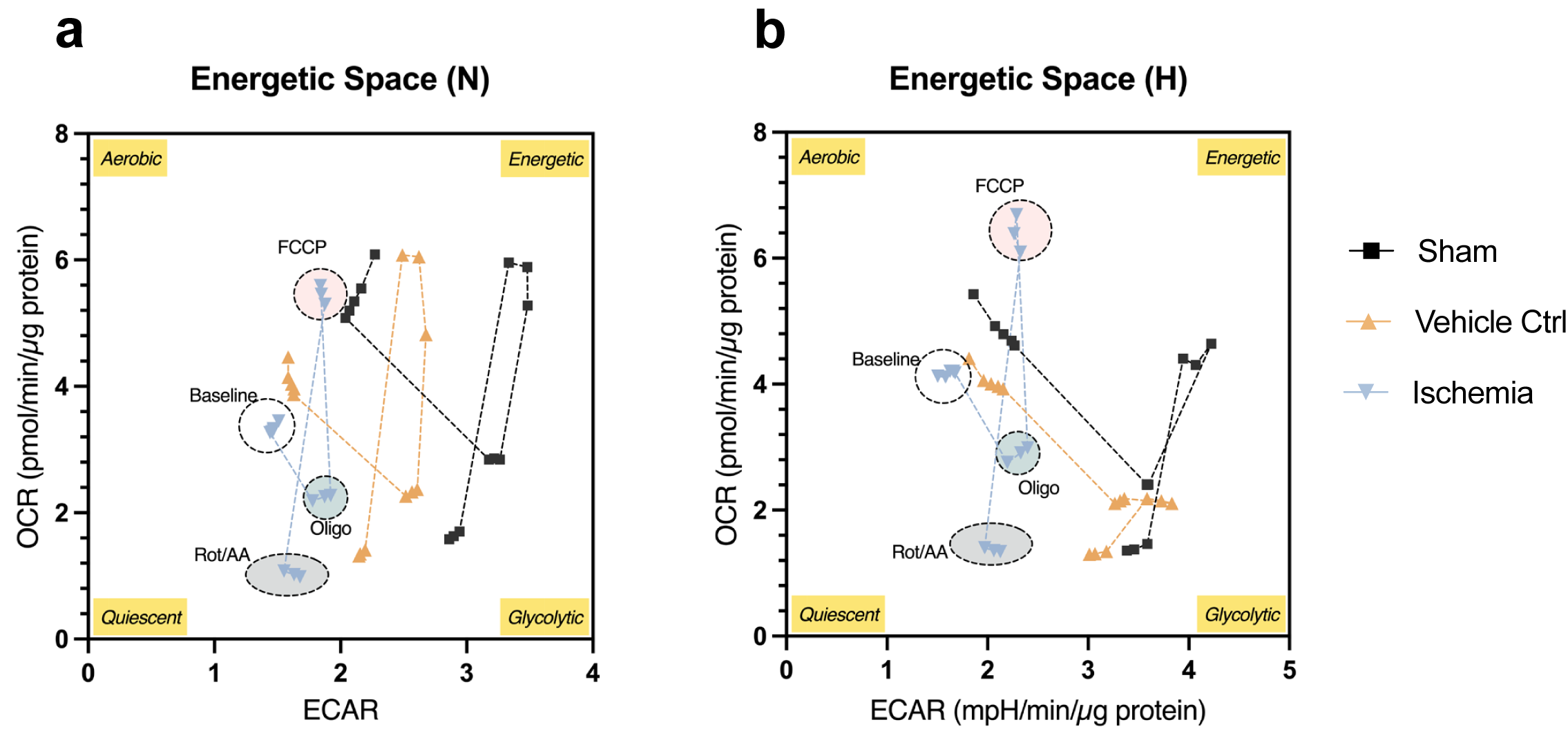

Supplement: Supplementary file 3 — Additional file 3. Figure S3. Bioenergetic alterations following ischemic conditioning in kidney proximal tubule cells. (a, b) Energetic space generated by plotting OCR vs ECAR levels before and after injections of oligomycin, FCCP, and rotenone/antimycin A. Data are shown as mean ± SD of ten replicates of three independent experiments. One-way ANOVA statistical analysis performed (*p value < 0.05; **p value < 0.01; ***p value < 0.001; ****p value < 0.0001). N, normoxia; H, Hypoxia. [file 13287_2023_3563_MOESM3_ESM.tif]

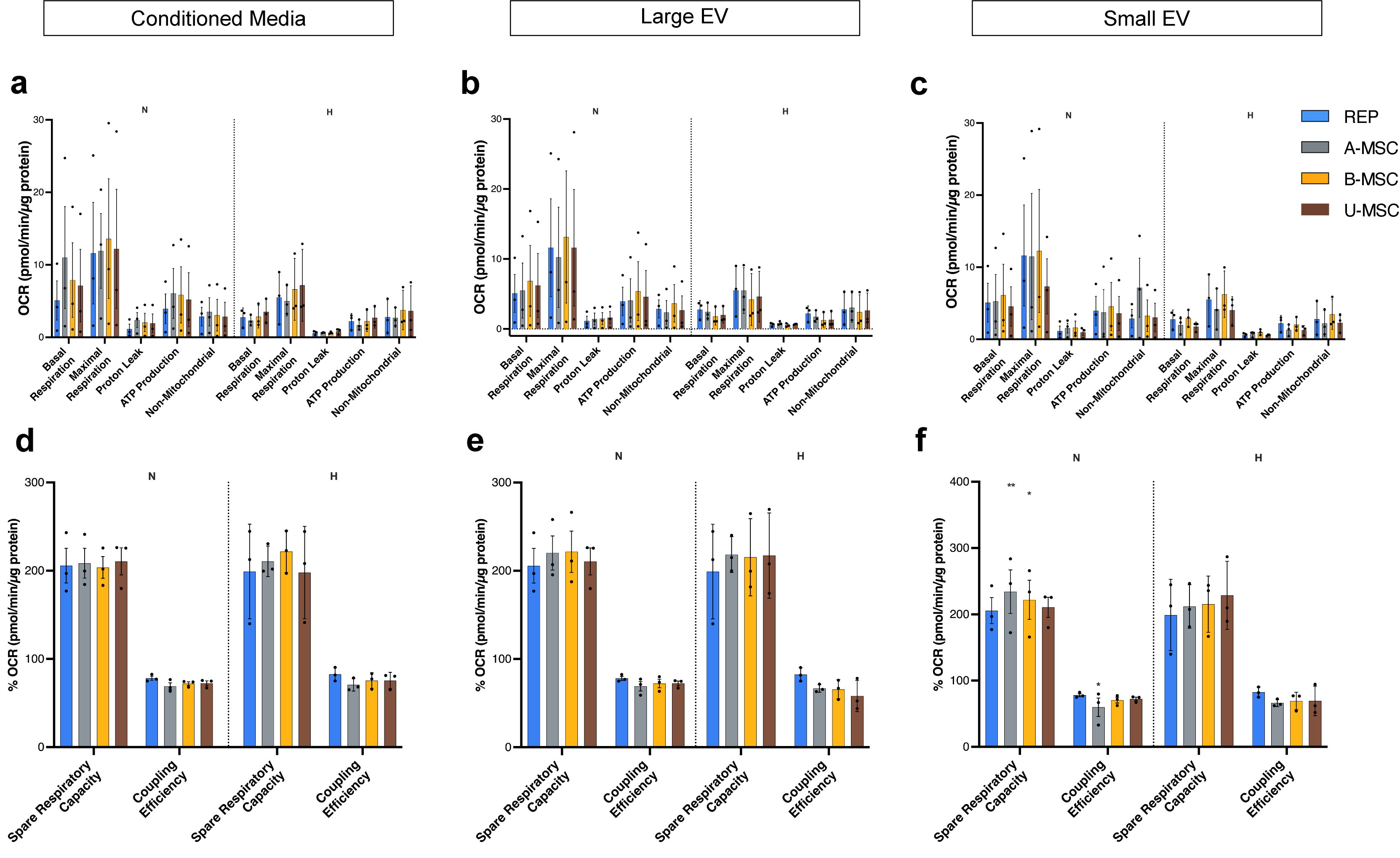

Supplement: Supplementary file 4 — Additional file 4. Figure S4. Breakdown of OCR-related parameters of MSC-treated N-ciPTECs and H-ciPTECs of CM (a,d), Large EVs (b,e), and Small EVs (c,f). Data are shown as mean ± SD of 6 replicates of three independent experiments. Statistical analysis performed using Two-Way ANOVA with Dunnett’s post-hoc test. *p value < 0.05; **p value < 0.01; ***p value < 0.001; ****p value < 0.0001). N, normoxia; H, hypoxia. [file 13287_2023_3563_MOESM4_ESM.tif]

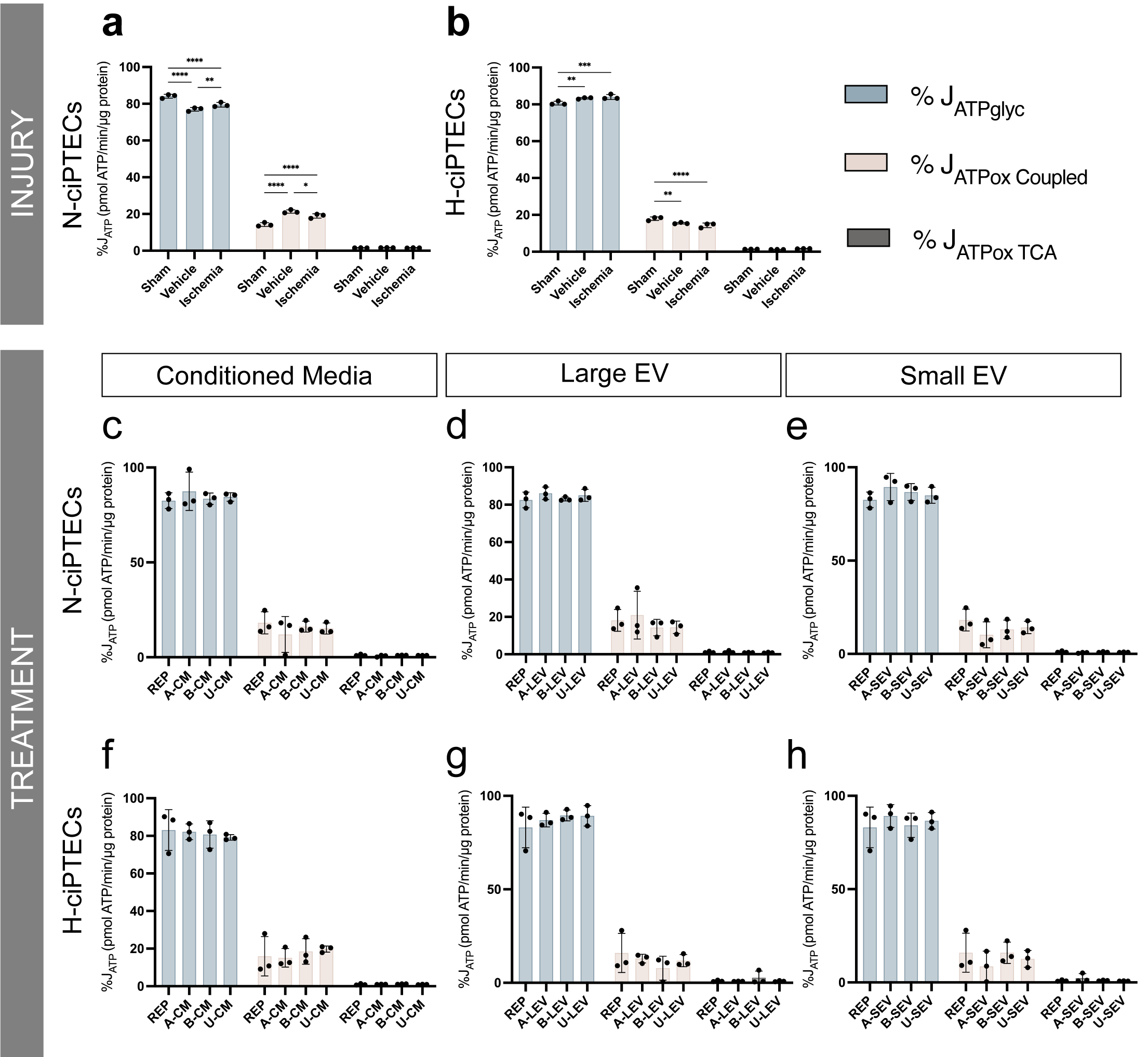

Supplement: Supplementary file 5 — Additional file 5. Figure S5. Net rate of ATP production (JATP) of ischemic ciPTECs (a,b), and ischemic ciPTECs treated with MSC secretome (c-h). The JATP divided in its three main components (JATPglyc, JATPox coupled, JATPox TCA). Statistical analysis performed using Two-way ANOVA and Dunnet’s post-hoc test (*p value < 0.05). Data are shown as mean ± SD of 6 replicates of three independent experiments. [file 13287_2023_3563_MOESM5_ESM.tif]

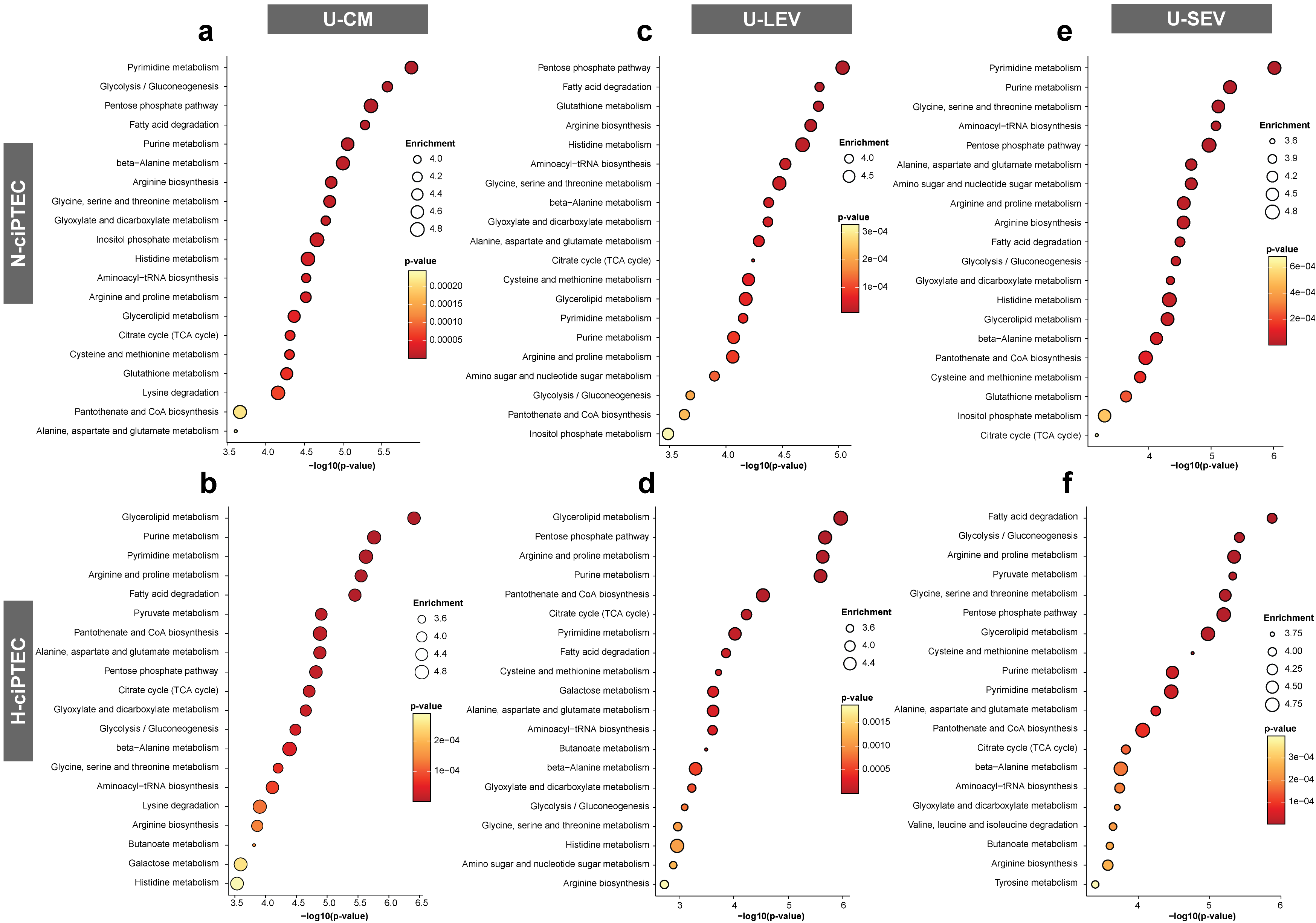

Supplement: Supplementary file 6 — Additional file 6. Figure S6. Metabolite sets enrichment overview showing the most altered metabolites revealed in ischemic ciPTECs treated with U-CM (a,b), U-LEV (c,d), or U-SEV (e–f), representing their physiological relevance. The top 25 metabolites for each treatment were compared to the reperfusion group (positive control). N, normoxia; H, hypoxia. [file 13287_2023_3563_MOESM6_ESM.tif]
