# Supplementary material for: Mesenchymal stromal cells secretome restores bioenergetic and redox homeostasis in human proximal tubule cells after ischemic injury
Source: Stem Cell Res Ther. 2023 Dec 10;14:353. doi: 10.1186/s13287-023-03563-6 (PMC10712181; doi:10.1186/s13287-023-03563-6)
Supplement: Supplementary file 7 — Additional file 7. Immunofluorescence analysis antibodies. [file 13287_2023_3563_MOESM7_ESM.docx]

**Supplementary Table**

**Supplementary Table S1.**Immunofluorescence analysis antibodies

| **Antibody** | **Species** | **Dilution** | **Catalog #** | **Company** |
| --- | --- | --- | --- | --- |
| Phalloidin |  | 1:1000 | A12381 | Invitrogen |
| Col IV | Goat | 1:50 | 134001 | SouthernBiotech |
| Alexa-488 goat anti-mouse | Goat | 1:500 | Ab150113 | Abcam |
| DAPI |  | 1:1000 | D3571 | ThermoFischer |
